# Household food insecurity among refugees: examining variations across different climatic contexts in Ugandan refugee settlements

**DOI:** 10.1080/16549716.2026.2702832

**Published:** 2026-07-22

**Authors:** Michael T. Wagaba, Christopher G. Orach, Alex Mulyowa, Maiya G. Block Ngaybe, Arthur Bagonza, Aggrey David Mukose, Justine Bukenya, Calvin Murungi, Hilde Bastiaens, Jean-Pierre Van Geertruyden

**Affiliations:** a Department of Community Health and Behavioural Sciences, Makerere University School of Public Health, Kampala, Uganda; b Department of Health Policy Planning and Management, Makerere University School of Public Health, Kampala, Uganda; c Department of Health Promotion Sciences, Mel and Enid Zuckerman College of Public Health, University of Arizona, Tucson, USA; d Department of Epidemiology and Biostatistics, Makerere University School of Public Health, Kampala, Uganda; e Department of Public Health, African Humanitarian Action, Kampala, Uganda; f Department of Family Medicine and Population Health, Faculty of Medicine and Health Sciences, University of Antwerp, Antwerp, Belgium

**Keywords:** Sustainable development, displaced populations, Uganda, public health, multisite study

## Abstract

**Background:**

Over a quarter of Uganda’s population is food-insecure. Agrarian refugee populations are disproportionately affected; however, evidence on how food insecurity varies across refugee settlements by climatic region remains limited.

**Objective:**

We assessed refugee household food insecurity across three settlements located in different climatic regions of Uganda.

**Methods:**

A cross-sectional study was conducted among 600 refugees from Kiryandongo, Nakivale, and Rhino Camp (200 per settlement). The Household Food Insecurity Access Scale was used to assess food insecurity and to compare it across and within settlements, using the adjusted chi-square test and post hoc survey-weighted tests. Modified Poisson regression identified factors associated with food insecurity, with 95% confidence intervals.

**Results:**

Household food insecurity was high (84.7%, 508/600) and varied significantly by settlement and background characteristics. Prevalence was highest among households in Rhino Camp settlement (100%, *p* < 0.001), situated within the unimodal rainfall region, among female-headed households (*p* = 0.009) and among households with heads aged 18–30 years (*p* = 0.02). Households with 7–12 members had a 10% higher prevalence compared with those with 1–6 members (APR = 1.10; 95% CI: 1.02–1.19). Conversely, food insecurity was 9% lower among households with married heads (APR = 0.91; 95% CI: 0.85–0.97), and 12% lower among households whose heads had secondary education or higher (APR = 0.88; 95% CI: 0.79–0.99).

**Conclusions:**

Food insecurity was more prevalent in unimodal rainfall regions, larger families, female-headed households, and those with older heads. In contrast, households with higher education levels were more food secure. Implementing gender-sensitive interventions and educational initiatives would be essential for improving food security.

## PAPER CONTEXT

- **Main findings:** The prevalence of food insecurity was significantly higher in Rhino Camp (36%) and Nakivale (21%) than in Kiryandongo settlement. Additionally, larger households, unmarried status, and lower educational attainment of household heads were also associated with increased food insecurity.
- **Added knowledge:** This paper contributes to a more nuanced understanding of the drivers of food insecurity in agrarian refugee settings across different geographic regions and the critical need for tailored interventions that account for the specific social, educational, and economic dynamics at play, while considering climate change and variability.
- **Global health impact for policy and action:** To combat food insecurity among refugee populations, particularly in remote agrarian areas, implementing gender-sensitive interventions and promoting education and skills-building initiatives in climate-sensitive agriculture and financial literacy are crucial. Policies should focus on promoting family planning to reduce household sizes, improving agricultural productivity and diversifying income sources beyond remittances and agriculture.

## Background

Food insecurity is a significant public health problem affecting almost a third of the global population [1]. By 2020, it was estimated that 11.7% of the global population faced severe food insecurity, while almost a third had inadequate access to food [2]. In Africa, more than 50% of the population was exposed to moderate to severe food insecurity [3]. East Africa also reported high levels of food insecurity on the continent, with 63% of its population affected [4]. Studies have shown that food insecurity is aggravated by climate change, particularly in low-resource settings. Rapid and uncertain changes in rainfall and temperature patterns threaten food production and could lead to an increase in food prices, reducing food accessibility and utilisation [5,6]. In Uganda, about 26% of the population experiences food insecurity, 5% of whom are vulnerable refugee populations already considered to be in the crisis phase [7].

Food insecurity is differentially experienced across refugee settlements in Uganda. These settlements are distributed across distinct agro-ecological zones, characterised by differing rainfall patterns and frequency of climate-related shocks such as droughts and floods [8–10]. These climatic variations influence agricultural productivity, and consequently, household food availability [11]. However, agricultural production in these settings is largely rain-dependent, heightening the differential vulnerability to food insecurity by climatic region. Therefore, this study used settlement locations across different climatic regions as a proxy. At the same time, non-climatic factors, in particular the socioeconomic and structural differences such as poor livelihoods and access to humanitarian aid among refugee households, limit their ability to purchase sufficient food, further heightening their vulnerability to food insecurity and hunger [11].

Despite existing evidence on the food insecurity crisis in the typically agrarian refugee settlements in Uganda, and the socioeconomic variations it exhibits, there remains a notable gap in understanding household food insecurity across the country’s different climatic regions. This study, therefore, assessed household food insecurity in three refugee settlements across different geographic regions of Uganda, each situated within a distinct climatic zone. The findings aim to inform context-specific strategies to strengthen resilience to climate change-aggravated food insecurity in refugee settings.

## Methods

### Study setting

**Figure 1.**
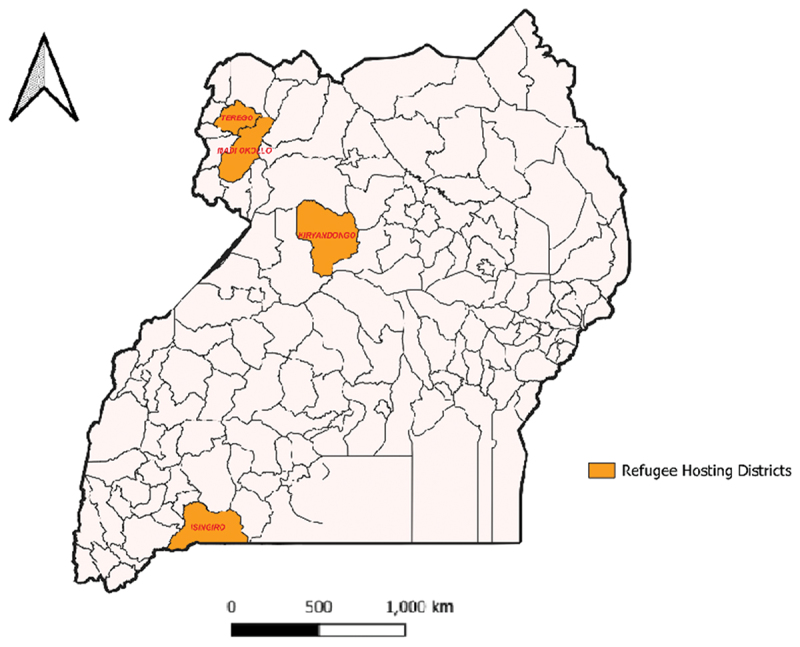
Map showing the locations of the different refugee settlements.

The study was conducted across three refugee settlements (Figure 1) in Uganda: Rhino Camp Refugee Settlement in the West Nile region (northwestern Uganda), Nakivale Refugee Settlement in the southwestern region, and Kiryandongo Refugee Settlement in the Western region. These settlements were purposively selected to represent the diverse climatic, geographic, and socio-economic contexts and experiences among the displaced populations. Rhino Camp refugee settlement, situated in the West Nile region near the South Sudan border, covers approximately 225 square kilometres and comprises 10 zones and 42 villages. It has a total population of 121,580 refugees and asylum seekers, consisting mainly of South Sudanese refugees [12]. It is characterised by an unimodal rainfall pattern, with one dry season and one rainy season [13].

Nakivale refugee settlement, one of Uganda’s oldest, is located in Isingiro District, near the border with Rwanda and the Democratic Republic of the Congo (DRC). It hosts an estimated 136,160 refugees from various countries, including Burundi, Somalia, Rwanda, and the DRC. Nakivale is a central reception centre since its inception in 1958 [12]. It is located in the wetlands and receives erratic rainfall [14].

Kiryandongo refugee settlement, situated in Kiryandongo district, was established in 2000 and is approximately 225 kilometres north of Kampala city, hosts 70,749 refugees predominantly South Sudanese, fleeing ongoing conflict in their homeland [12]. Kiryandongo’s weather is characterised by a bimodal rainfall pattern, influencing livelihood strategies and community dynamics among its predominantly agricultural population [15].

## Study design

The study employed a cross-sectional design with a quantitative approach.

## Study population

The study population comprised refugees in the three selected settlements (Kiryandogo, Nakivale, and Rhino). Household heads who had resided within the settlements for at least 10 years participated in the study. This criterion was considered to elicit responses inherently informed by cumulative food security changes, resonating with climatic changes that occur over long periods [16]. However, this approach left out newly registered refugees, who are also prone to insecurity. We excluded participants who were absent from their homes at the time of data collection and those who did not consent to participate.

## Sample size determination

We used Yamane’s formula to estimate the sample size [17]. Considering the total population across the three settlements: Nakivale (136,160), Kiryandongo (70,749), and Rhino Camp (121,580), a margin of error of 5% and a design effect of 1.5, we attained a sample size of 600 participants [12]. We then applied a stratified disproportionate distribution to allocate an equal sample of 200 respondents to each settlement. A disproportionate distribution method was employed to ensure that each settlement was adequately represented despite differences in population size and demographics [18,19].

## Sampling procedure

Three settlements were purposively chosen from the 13 refugee settlements in Uganda to ensure a diverse representation of climatic, geographic, ethnic, and socio-economic contexts among the refugees. In the initial stage, we obtained a list of villages within each settlement from the Office of the Prime Minister (OPM) and UNHCR. From this list, we randomly selected five villages from each settlement, totaling 15 villages. This selection process utilised simple random sampling techniques: the names of the villages were written on slips of paper, mixed, and drawn without replacement. The decision to select five villages was driven by logistical considerations, including resource limitations, accessibility to settlements, and the need for geographic representation across the settlements. In the second stage, household selection was conducted within the sampled villages. Given that the existing ProGres database, used by OPM and UNHCR, contained inaccuracies because it erroneously represented households that had migrated to parts of Uganda outside the settlements, a household listing was conducted. With the support of the refugee settlement leaders, the data collection team compiled updated lists of households that met the inclusion criteria to develop a sampling frame while in the field. From these lists, eligible households were selected using simple random sampling, through the use of random number generators. All eligible households had representatives present at the time of data collection, as local leaders had helped inform and mobilise community members.

## Data collection procedures

The data were collected in March 2023. Three teams, each comprising five research assistants fluent in the local languages spoken in their respective refugee settlements, were deployed to collect the data. The teams administered structured questionnaires, via the KoboCollect tool, to collect data from respondents. The RAs were trained in proper data collection techniques, ethical considerations, study protocols, and the use of the KoboCollect tool, which was specifically designed for the study. Two principal investigators and three study coordinators supervised data collection and collated it for quality assurance.

## Study variables

The dependent variable for this study was food insecurity. Food insecurity was assessed using the Household Food Insecurity and Access Scale (HFIAS) [20,21]. The HFIAS is a standardised survey developed by the United Nations Food and Agriculture Organisation (FAO) to measure the extent and severity of food insecurity at the household level. The HFIAS questionnaire comprises nine questions about household food consumption and access to food over the past month [21]. Respondents were asked nine questions about their access to adequate food in the past 30 days. The questions were designed to capture the various dimensions of food insecurity, including the quantity, quality, and variety of food consumed, as well as the psychological and social aspects of food insecurity. A score was developed for each household, and households were grouped into various categories of food insecurity. The HFIAS has four outcome categories: secure, mildly food-insecure, moderately food-insecure, and severely food-insecure. However, we dichotomised the four categories by grouping the secure and mildly insecure categories as ‘food secure’, and the moderately and severely insecure as ‘food insecure’. In Ugandan refugee settlement contexts, mild food insecurity could be subjectively regarded as food secure [22,23].

The independent variables included socio-demographic factors such as gender, age, education level, household size, marital status, length of stay in the settlement, settlement location, and primary source of household income. To select the independent variables, we considered information from implementing partners and factors previously identified in prior studies as significantly associated with refugee food insecurity.

## Data management and analysis

Data cleaning was performed using Microsoft Excel. We computed and applied sampling weights to account for unequal selection probabilities among participants in the three refugee settlements. This weight was computed as the probability of selecting individuals aged 18 years or older in each settlement [24], relative to the total population across the three settlements. It was then applied at the household level, assuming that each sampled household was represented by an adult household head. This sampling weight was applied to all descriptive and inferential analyses performed.

Analysis was performed using STATA version 16 [25]. We performed univariate analyses to describe respondents’ sociodemographic characteristics and the overall proportion of households experiencing food insecurity, and stratified by background characteristics. Differences between variable categories were determined using the Rao–Scott adjusted chi-square test. Post hoc statistical differences were assessed using survey-weighted tests of predicted probabilities. All independent variables were tested for collinearity at a significance value of 0.4; however, no variables were found to be significantly related to one another. To build the multivariable model, all variables were included, even those with *p*-values > 0.05 in the bivariate analyses, given their established relationships with food insecurity. Potential confounders included in the model were the household head’s age and sex. Significantly associated factors with food insecurity were identified using a modified Poisson regression model, built via forward inclusion and backward elimination of all independent variables at a 95% confidence level and a 5% significance level.

## Ethical considerations

Ethical clearance for the study was granted by the Makerere University School of Public Health Research and Ethics Committee (SPH-2023–488) and the Uganda National Council for Science and Technology (HS3685ES). Permission to collect data within refugee settlements was also obtained from the Office of the Prime Minister (OPM), Department of Refugees, Kampala. All respondents provided written informed consent before participating in the study. The data collection took place at the respondents’ residences in the settlements. This ensured the safety, privacy, and anonymity of the study participants and research assistants throughout the data collection process.

## Results

### Sociodemographic characteristics of the respondents

More than half (59.4%, *n* = 345) of the respondents were aged 31–54; 3 out of 5 (60.6%, *n* = 372) were female; almost half (49.0%) were South Sudanese; and two-thirds (66.1%, *n* = 391) were married. The majority (80.5%, *n* = 468) of the respondents had only attained primary education or lower, and most (34.5%) relied on remittances as their primary source of income. About half (48.9%, *n* = 284) of the households had 7–12 members, and almost two-thirds (66.1%) were married (Table 1).

**Table 1.** Sociodemographic characteristics of the respondents.

Variable		Frequency (n = 600)	Percentage (%)
Age-group (years)	18–30	171	25.4
	31–54	345	59.4
	55+	84	15.2
Gender	Male	228	39.4
	Female	372	60.6
Household size	1–6	242	43.5
	7–12	284	45.9
	13+	74	10.6
Years lived in the settlement	10–19	457	76.2
	20–29	106	17.7
	30+	37	6.2
Marital status	Not married	209	33.9
	Married	391	66.1
Education level	Primary and below	468	80.5
	Secondary and above	132	19.5
Primary source of household income	Begging	9	2.0
	Remittances	245	34.5
	Salary/wages	30	5.1
	Non-agri-business	38	7.8
	Agriculture	272	49.5
	Others	6	1.1
Ethnicity	South Sudanese	359	49.0
	Congolese	118	24.3
	Rwandans	76	17.0
	Burundi	39	8.5
	Somali	2	0.4
	Others	6	0.8

### Prevalence of refugee household food insecurity, overall and by background characteristics

Table 2 depicts that the prevalence of food insecurity was high (84.7%, 508/600). Food insecurity levels varied across settlements, with the highest levels in Rhino Camp; all respondents (100%, *n* = 200) were food insecure.

**Table 2.** Food security status, overall and by settlement.

HFIAS Category		Settlement		
	Overall (n, %)	Kiryandongo (n, %)	Nakivale (n, %)	Rhino Camp (n, %)
Food secure	59 (9.8)	46 (23.0)	13 (6.5)	0 (0.0)
Mildly food insecure	33 (5.5)	16 (8.0)	17 (8.5)	0 (0.0)
Moderately food insecure	69 (11.5)	37 (18.5)	32 (16.0)	0 (0.0)
Severely food insecure	439 (73.2)	101 (50.5)	138 (69.0)	200 (100.0)
**Dichotomised food security status**				
Food secure	92 (15.3)			
Food insecure	508 (84.7)			

Further variations were observed across different socio-demographic characteristics, including household size, marital status, and educational level. In multivariable analysis, the prevalence of food insecurity was 36% higher in Rhino Camp (APR = 1.36; 95% CI: 1.18–1.57) and 21% higher in Nakivale (APR = 1.21; 95% CI: 1.01–1.45) than in the Kiryandongo refugee settlement. The prevalence of food insecurity was also 10% higher among households with 7–12 members (APR = 1.10; 95% CI: 1.02–1.19) than among those with fewer than 7 members. Being married was also associated with a 9% decrease in the prevalence of food insecurity (APR = 0.91; 95% CI: 0.85–0.97) as compared to respondents who were not married. Additionally, households in which the household head had attained secondary education or higher, had a 12% lower prevalence of food insecurity (APR = 0.88; 95% CI: 0.79–0.99) than those with a primary education or lower (Table 3).

**Table 3.** Prevalence of household food insecurity with overall differences and associations by background characteristics.

Variable	Category	Food secure n (%)	Food insecure n (%)	F-test *p*-value	95% CI	post-hoc *p*-value	aPR (95% CI)
Food security status	Overall	92 (15.5)	508 (84.7)				
Refugee settlement	Kiryandongo	62 (31.0)	138 (69.0)		(62.2–75.1)		1.00
	Nakivale	30 (15.0)	170 (85.0)	**p < 0.001**	(79.3–89.3)	p < 0.001	1.21 (1.01–1.45)*
	Rhino Camp	0	200 (100.0)		0		1.36 (1.18–1.57)*
Sex	Male	45 (20.4)	183 (79.6)	**0.009**	(73.6–84.5)		1.00
	Female	47 (12.2)	325 (87.8)		(84.1–90.7)		1.02 (0.94–1.10)
Age-group	18–30	13 (8.2)	158 (91.8)	**0.02**	(86.4–95.2)	0.003	1.00
	31–54	64 (18.3)	281 (81.7)		(77.2–85.5)		0.99 (0.92–1.07)
	55+	15 (16.6)	69 (83.4)		(73.8–89.9)		1.00 (0.89–1.13)
Household size	1–6	53 (21.0)	189 (79.0)	**0.003**	(73.3–83.7)	0.003	1.00
	7–12	29 (10.3)	255 (89.7)		(85.4–92.8)		1.10 (1.02–1.19)*
	13+	10 (14.8)	64 (85.2)		(74.5–91.9)		1.03 (0.91–1.16)
Years lived in the settlement	10–19	64 (14.7)	393 (85.3)	0.16	(81.7–88.3)		1.00
	20–29	18 (15.1)	88 (84.9)		(76.9–90.4)		1.05 (0.93–1.18)
	30+	10 (27.4)	27 (72.6)		(55.9–84.8)		0.99 (0.79–1.24)
Marital status	Not married	26 (12.2)	183 (87.8)	0.13	(82.5–91.6)		1.00
	Married	66 (17.1)	325 (82.9)		(78.8–86.4)		0.91 (0.85–0.97)*
Highest completed level of education	Primary and lower	58 (12.4)	410 (87.6)	**0.0001**	(83.9–90.1)		1.00
	Secondary and higher	34 (25.8)	98 (74.2)		(64.7–80.1)		0.88 (0.79–0.99)*
Primary source of household income	Remittances	11 (5.0)	234 (95.0)	**p < 0.001**	(91.1–97.2)	0.003^a^ 0.025^b^ p < 0.001^c^	1.00
	Salary/wages	13 (36.9)	17 (63.1)		(45.1–78.1)		0.77 (0.57–1.02)
	Non-agri-business	11 (27.7)	27 (72.3)		(56.0–84.3)		0.84 (0.67–1.06)
	Begging	0 (0.0)	9 (100.0)		0		1.08 (0.91–1.27)
	Agriculture	56 (18.6)	216 (81.4)		(76.4–85.5)		0.96 (0.83–1.11)
	Others	1 (21.0)	5 (78.9)		(30.0–97.0)		0.80 (0.53–1.20)
Ethnicity	Burundi	7 (17.9)	32 (82.1)	**0.0003**	(65.6–90.8)		1.00
	Congolese	21 (17.8)	97 (82.2)		(73.9–88.1)		0.99 (0.83–1.17)
	Rwandans	8 (10.5)	68 (89.5)		(81.5–95.2)		1.05 (0.88–1.25)
	South Sudanese	50 (13.9)	309 (86.1)		(81.1–88.2)		1.00 (0.81–1.23)
	Others	6 (75.0)	2 (25.0)		(2.2–63.3)		0.35 (0.10–1.21)

^a^salary vs remittances.

^b^non-agribusiness vs remittances.

^c^agriculture vs remittances.

Settlement-stratified analyses demonstrated that the relationship between background characteristics and household food insecurity varied across settlements, with more pronounced differences observed in Kiryandongo (Table 4). All Rhino Camp respondents were food insecure, so no binary regression was possible. In Kiryandongo, households with 7–12 members had a 28% higher prevalence of food insecurity compared to those with fewer than 7 members (APR = 1.28; 95% CI: 1.02–1.59). Conversely, households with married heads had a 16% lower prevalence of food insecurity than those with unmarried heads (APR = 0.84; 95% CI: 0.70–0.99). Households whose primary source of income was ‘salary or wages’ showed a remarkable 76% lower prevalence of food insecurity (APR = 0.24; 95% CI: 0.06–0.87). In addition, South Sudanese and Congolese households had lower prevalence of food insecurity at 35% and 39%, respectively (APR = 0.65; 95% CI: 0.50–0.86; APR = 0.61; 95% CI: 0.40–0.93).

**Table 4.** Prevalence of household food insecurity by background characteristics, showing associations per settlement.

			Food insecurity status across the settlements				
		Food insecurity status overall	Kiryandongo		Nakivale		**Rhino Camp
Variable	Category	(*N* = 508)	(*n* = 138)	aPR (95% CI)	(*n* = 170)	aPR (95% CI)	(*n* = 200)
Sex							
	Female	325	79 (20.6)	1	100 (42.9)	1	146 (36.4)
	Male	183	59 (26.1)	1.20 (0.98–1.47)	70 (51.0)	0.88 (0.77–1.00)	54 (22.9)
Age-group							
	18–30	158	8 (4.8)	1	24 (23.5)	1	126 (71.7)
	31–54	281	104 (29.7)	1.56 (0.91–2.69)	111 (52.2)	0.89 (0.77–1.03)	66 (18.1)
	55+	161	26 (28.5)	1.55 (0.88–2.74)	35 (63.1)	0.92 (0.78–1.09)	8 (8.4)
Household size							
	1–6	189	44 (17.8)	1	94 (62.5)	1	51 (19.7)
	7–12	255	74 (24.9)	1.28 (1.02–1.59)*	72 (39.9)	1.11 (0.97–1.27)	109 (35.1)
	13+	64	20 (30.8)	1.18 (0.85–1.62)	4 (10.2)	0.78 (0.46–1.31)	40 (59.0)
Years lived in the settlement							
	10–19	393	80 (17.5)	1	114 (41.0)	1	199 (41.5)
	20–29	88	34 (27.7)	1.01 (0.81–1.27)	54 (72.3)	1.03 (0.89–1.19)	0 (0.0)
	30+	27	24 (85.0)	0.94 (0.71–1.23)	2 (11.6)	0.82 (0.43–1.59)	1 (3.4)
Marital Status							
	Not married	183	61 (28.5)	1	53 (40.7)	1	69 (30.8)
	Married	325	77 (19.5)	0.84 (0.70–0.99)*	117 (48.8)	0.94 (0.83–1.06)	131 (31.7)
Highest completed level of education							
	Primary and below	410	104 (20.5)	1	159 (51.7)	1	147 (27.8)
	Secondary and above	98	34 (33.1)	0.83 (0.65–1.06)	11 (17.6)	0.71 (0.49–1.03)	53 (49.3)
Primary source of household income							
	Remittances	234	34 (14.7)	1	9 (6.4)	1	191 (78.9)
	Salary/wages	17	2 (7.5)	0.24 (0.06–0.87)*	15 (92.5)	1.14 (0.80–1.61)	0 (0.0)
	Non-agri-business	27	4 (9.9)	0.69 (0.35–1.35)	21 (85.4)	0.97 (0.69–1.37)	2 (4.7)
	Begging	9	1 (7.1)	1.32 (0.88–1.98)	8 (92.9)	1.29 (0.94–1.76)	0 (0.0)
	Agriculture	216	97 (33.4)	0.85 (0.68–1.05)	115 (65.2)	1.09 (0.83–1.43)	4 (1.3)
	Others	5	0 (0.0)		2 (53.4)	0.80 (0.37–1.74)	3 (46.6)
Ethnicity	Burundi	32	3 (6.0)	1	28 (92.1)	1	1 (1.9)
	South Sudanese	309	117 (38.7)	0.65 (0.50–0.86)*	3 (1.6)	1.54 (1.01–2.34)*	189 (59.7)
	Congolese	97	14 (9.7)	0.61 (0.40–0.93)*	73 (83.6)	0.97 (0.80–1.16)	10 (6.7)
	Rwandans	68	2 (1.8)	0.56 (0.24–1.33)	66 (98.2)	1.06 (0.89–1.28)	0 (0.0)
	Others	2	2 (100.0)	0.30 (0.10–0.85)	0 (0.0)	0	0 (0.0)

The percentages used are weighted row percentages.

***Rhino = All respondents from Rhino Camp settlements reported to be severely food insecure, and hence no further binary regression could be performed.

In contrast, in Nakivale, significant variation in food insecurity was observed only by ethnicity. Refugees of South Sudanese origin had a 54% higher prevalence of food insecurity (APR = 1.54; 95% CI: 1.01–2.34) compared to other origins.

## Discussion

This study aimed to assess levels of household food insecurity among refugees living in three distinct settlements, each characterised by different geographic, demographic, and climatic conditions. The study found that household food insecurity was notably high and varied across the settlements. The groups most affected included those in Rhino Camp, female-headed households, households with 7–12 members, and South Sudanese refugees in Nakivale. Conversely, refugee households in which the head of the family was married, had attained secondary education or higher, experienced lower levels of food insecurity. Households in Kiryandongo, where the primary source of income for the household head was salary or wages, also experienced lower levels of food insecurity.

The finding that refugees experience significant levels of food insecurity within the three settlements studied aligns with existing local and global literature, which has reported almost similar high levels of food insecurity among refugees and other displaced populations [26–28]. Such issues are often linked to poor livelihoods and low socioeconomic status [29,30]. Moreover, the food insecurity could have been aggravated by the adverse impacts of climate change on agricultural production in these socioeconomically marginalised communities [31]. The findings may also be attributed to the ongoing influx of refugees, leading to increased competition for land and other resources [32,33]. The growing refugee populations heighten food demand, placing additional strain on food systems, which in turn affects food quality, quantity, and equitable distribution [34]. Our findings, therefore, underscore the need for policy and program responses that address food needs and structural vulnerabilities of refugees in relation to food insecurity.

Rhino Camp exhibited the highest prevalence of food insecurity as compared to Nakivale and Kiryandongo. Rhino Camp, located in the West Nile region, is characterised by a unimodal rainfall pattern, while Kiryandongo in the mid-west and Nakivale in the south-west experience bi-modal rainfall patterns. As a result, Rhino Camp experiences prolonged droughts that exacerbate the food scarcity. These findings are consistent with existing literature that highlights disparities in food insecurity levels based on geographical location [35,36]. The heightened food scarcity is known to increase market prices and strain food accessibility, utilisation, and stability for the population [37]. These findings highlight how geographical location and climatic conditions impact food security outcomes. They emphasise the need to integrate climate change adaptation strategies into efforts to combat food insecurity in refugee settlements, with careful attention to the specific geographic contexts.

Food insecurity was associated with certain ethnic groups in varying settlement locations. For example, South Sudanese refugees who settled in Nakivale were more likely to be food insecure than those in Kiryandongo. While this finding may warrant further inquiry, plausible explanations could be offered. First, different ethnic groups usually have unique foods grown, agricultural practices, food taboos and preferences, and storage practices. These differences can shape how households produce, obtain, consume, and sustain food [38]. In addition, ethnic differences which are linked to variations in social, cultural, economic, and lifestyle patterns may influence the dimensions of food security, i.e. availability, access, utilisation, and stability [39,40]. Second, ethnic background and subsequent integration into the settlement context may also influence the vulnerability of refugees to food insecurity and their adaptation mechanisms. This could be particularly important when considering how climate variability affects food production across different geographical areas. Nonetheless, further research is warranted to explore this possible relationship between settlement context, ethnicity, and food insecurity.

The study found that female-headed households reported higher levels of food insecurity, highlighting a significant gender disparity in vulnerability within refugee settings. Similar observations have been reported in previous research, which indicates that female-headed households are more likely to experience food insecurity [41]. This trend may stem from differential gendered privileges within these communities, in which women face considerable challenges in accessing food due to unequal economic opportunities and limited decision-making power [42]. Various studies have emphasised the crucial role that women play in fostering food security and enhancing the household social well-being, particularly within refugee communities [43,44]. The identified gender-based disparities could highlight a lack of gender-sensitive approaches in mitigating food insecurity in agrarian refugee settlements.

Another aspect of differential vulnerability to food insecurity in the refugee settlement context was variability in education levels. The findings indicated that food insecurity was significantly lower among respondents who had attained secondary education or higher. Similar studies conducted in Uganda and Kenya corroborate this trend, highlighting the essential role of education in enhancing food production, access, utilisation, and sustainability [26,45]. Educated community members tend to have better employment opportunities, which, in turn, lead to higher incomes, enabling them to meet basic needs, including sufficient food [46]. Furthermore, higher educational attainment often leads to greater access to information about improved agricultural practices, food utilisation, and storage [47]. The practical application of this knowledge can bolster resilience to food insecurity in refugee settlements in remote, marginalised settings that are disproportionately exposed to the adverse effects of climate change. Therefore, it is imperative to implement and examine interventions that incorporate education and awareness programs to improve food security among refugee populations.

The study further indicates that in Rhino Camp, the intensity of food insecurity increases with household size. This finding corroborates the existing literature, which shows that larger households frequently experience heightened food insecurity due to intensified competition among members for finite resources [48,49]. Evidence indicates that refugee households usually have larger household sizes [50]. Perhaps, with larger household sizes, intra-household allocation for resources becomes a challenge, especially where expenditures could be diverted to non-essential items such as alcohol at the expense of food and other basic needs [51]. Awareness creation through sensitisation campaigns on prioritising household basic needs, such as food, may be critical to mitigate food insecurity in such contexts.

Our findings highlight the impact of a household’s income source on food insecurity. Households primarily dependent on salaries or wages reported the lowest prevalence of food insecurity, whereas those relying on remittances or agricultural income reported higher levels of food insecurity. This latter finding contradicts other studies that indicated an improved food security status among households that depended on remittances [50,52]. This difference in outcomes could also be explained by the societal contexts and beliefs described in the preceding paragraph. Agriculture, the alternative primary source of income, is less productive due to the small plots of land allocated to each refugee household and to unpredictable weather conditions. These low-income sources are associated with low purchasing power and diversification, which negatively impact food accessibility and utilisation [53].

## Strengths and limitations

The study offers new insights into food insecurity in remote agrarian refugee settlements in Uganda, focusing on how sociodemographic factors and geographic locations influence this issue. It compares the varying impacts of these factors on food insecurity across different refugee contexts, particularly considering their differing exposure to the adverse effects of climate change.

However, the dichotomisation of the HFIAS categories could have potentially lost analytical precision and granularity of the findings. In addition, the findings remain exploratory due to limitations in sample size. Also, the inclusion criterion requiring more than 10 years of residence could have limited generalisability and as well, the purposive selection of three settlements, potentially limited external validity. Furthermore, the use of settlement-level geographic and climatic regions as a proxy for climate exposure could have limited the ability to draw precise conclusions and generalisations. Nevertheless, we thoroughly explained the study’s rationale and emphasised the importance of providing honest responses before conducting the interviews.

Additionally, there could have been potential for selection bias in recruiting respondents given that the sampling frame was developed with the assistance of local leaders. However, we minimised this by ensuring that all households were selected completely at random. Moreover, the inclusion criteria required household heads to have lived in the settlement for at least 10 years to capture long-term exposure to climate changes, while food insecurity was assessed over the past 30 days. However, this short-term measure (HFIAS) may reflect underlying vulnerability linked to persistent structural and environmental changes over time. Thus, households facing food insecurity are likely affected by long-term contextual factors, including cumulative climate-related changes.

## Conclusions

Food insecurity was high among the surveyed households, but was highest in Rhino Camp, which is characterised by a unimodal rainfall pattern and is further associated with households with poor socio-economic attributes, such as low education and income levels. This underscores the need for targeted and context-specific interventions, livelihoods support, gender-sensitive programming, and education or relevant skills building within the context of climate variability among agrarian refugee settlements.

## Supplementary Material

Supplementary file_STROBE checklist.docx

## Data Availability

The data that support the findings of this study are available from the corresponding author, [MTW], upon reasonable request.
